# JAK Inhibition in Aicardi-Goutières Syndrome: a Monocentric Multidisciplinary Real-World Approach Study

**DOI:** 10.1007/s10875-023-01500-z

**Published:** 2023-05-12

**Authors:** Marie-Louise Frémond, Marie Hully, Benjamin Fournier, Rémi Barrois, Romain Lévy, Mélodie Aubart, Martin Castelle, Delphine Chabalier, Clarisse Gins, Eugénie Sarda, Buthaina Al Adba, Sophie Couderc, Céline D’ Almeida, Claire-Marine Berat, Chloé Durrleman, Caroline Espil, Laetitia Lambert, Cécile Méni, Maximilien Périvier, Pascal Pillet, Laura Polivka, Manuel Schiff, Calina Todosi, Florence Uettwiller, Alice Lepelley, Gillian I. Rice, Luis Seabra, Sylvia Sanquer, Anne Hulin, Claire Pressiat, Lauriane Goldwirt, Vincent Bondet, Darragh Duffy, Despina Moshous, Brigitte Bader-Meunier, Christine Bodemer, Florence Robin-Renaldo, Nathalie Boddaert, Stéphane Blanche, Isabelle Desguerre, Yanick J. Crow, Bénédicte Neven

**Affiliations:** 1 Paediatric Haematology-Immunology and Rheumatology Unit, Necker Hospital, APHP Centre, Université Paris Cité, 149 rue de Sèvres, 75015 Paris, France; 2 Imagine Institute, Laboratory of Neurogenetics and Neuroinflammation, Inserm UMR 1163, Université Paris Cité, 24 boulevard du Montparnasse, 75015 Paris, France; 3 Paediatric Neurology Department, Necker Hospital, APHP Centre, Université Paris Cité, 75015 Paris, France; 4 Department of Paediatric Rheumatology, Sidra Medicine, Doha, Qatar; 5 Neonatal Department, Poissy Saint-Germain Hospital, Poissy, France; 6 Paediatrics Department, Castres-Mazamet Intercommunal Hospital, Castres, France; 7 Reference Center of Inherited Metabolic Disorders, Necker Hospital, APHP, Université Paris Cité, 75015 Paris, France; 8 Paediatric Neurology Department, Bordeaux University Hospital, Bordeaux, France; 9 Genetics Department, Nancy University Hospital, 54000 Nancy, France; 10 Paediatric Dermatology Department, Necker Hospital, APHP Centre, Université Paris Cité, 75015 Paris, France; 11 Paediatric Neurology Department, Tours University Hospital, Tours, France; 12 Paediatric Rheumatology Department, Bordeaux University Hospital, Bordeaux, France; 13 Imagine Institute, Inserm UMR 1163, 75015 Paris, France; 14 Paediatric Neurology Unit, Children’s Medicine Department, Children’s Hospital, Nancy University Hospital, 54000 Nancy, France; 15 Paediatric Rheumatology Department, Tours University Hospital, Tours, France; 16 Division of Evolution and Genomic Sciences, School of Biological Sciences, Faculty of Biology, Medicine and Health, University of Manchester, Manchester Academic Health Science Centre, Manchester, UK; 17 Biochemistry, Metabolomics and Proteomics Department, Necker Hospital, AP-HP Centre, Université Paris Cité, 75015 Paris, France; 18 Pharmacology and Toxicology Laboratory, Henri Mondor University Hospital, APHP, 94000 Créteil, France; 19 Pharmacology Department, Saint-Louis University Hospital, APHP, 75010 Paris, France; 20 Translational Immunology Unit, Institut Pasteur, Université de Paris Cité, F75015 Paris, France; 21 Paediatric Neurology Department, Trousseau Hospital, APHP, Sorbonne Université, 75012 Paris, France; 22 Paediatric Radiology Department, AP-HP, Hôpital Necker Enfants Malades, Université Paris cité, Institut Imagine INSERM U1163 and U1299, 75015 Paris, France; 23 MRC Human Genetics Unit, Institute of Genetics and Cancer, Edinburgh, UK; 24 Imagine Institute, Laboratory of Immunogenetics of Paediatric Autoimmunity, INSERM UMR 1163, Université Paris Cité, 75015 Paris, France

**Keywords:** Aicardi-Goutières syndrome (AGS), interferon, JAK inhibitors

## Abstract

The paradigm type I interferonopathy Aicardi-Goutières syndrome (AGS) is most typically characterized by severe neurological involvement. AGS is considered an immune-mediated disease, poorly responsive to conventional immunosuppression. Premised on a chronic enhancement of type I interferon signaling, JAK1/2 inhibition has been trialed in AGS, with clear improvements in cutaneous and systemic disease manifestations. Contrastingly, treatment efficacy at the level of the neurological system has been less conclusive. Here, we report our real-word approach study of JAK1/2 inhibition in 11 patients with AGS, providing extensive assessments of clinical and radiological status; interferon signaling, including in cerebrospinal fluid (CSF); and drug concentrations in blood and CSF. Over a median follow-up of 17 months, we observed a clear benefit of JAK1/2 inhibition on certain systemic features of AGS, and reproduced results reported using the AGS neurologic severity scale. In contrast, there was no change in other scales assessing neurological status; using the caregiver scale, only patient comfort, but no other domain of everyday-life care, was improved. Serious bacterial infections occurred in 4 out of the 11 patients. Overall, our data lead us to conclude that other approaches to treatment are urgently required for the neurologic features of AGS. We suggest that earlier diagnosis and adequate central nervous system penetration likely remain the major factors determining the efficacy of therapy in preventing irreversible brain damage, implying the importance of early and rapid genetic testing and the consideration of intrathecal drug delivery.

**Supplementary Information:**

The online version contains supplementary material available at 10.1007/s10875-023-01500-z.

## Introduction

The paradigm type I interferonopathy Aicardi-Goutières syndrome (AGS) encompasses 9 genotypes (AGS1-9), proposed to share a common pathophysiology related to aberrant nucleic acid processing or sensing, with subsequent chronically enhanced activation of type I interferon signaling [1]. While the neurological phenotype of AGS is broad, the disease most frequently presents as an early-onset acute encephalitis, in some cases after several months of completely normal development. The encephalopathic period usually lasts several months, characterized by significant neurological irritability and a loss of previously acquired skills. Notably, the acute disease phase is often, albeit not always, followed by clinical stabilization with no apparent further disease progression, and with the acquisition of new, even if limited, milestones in some patients [2]. Mutations in *ADAR1* represent a special case, sometimes presenting with the subacute onset of bilateral striatal necrosis and severe dystonia [3].

Although not formally assessed, AGS is considered to be poorly responsive to conventional immunosuppressive therapies [4]. Based on the retroelement hypothesis of disease pathogenesis [5], Rice et al conducted a clinical trial of nucleoside reverse transcriptase inhibitors (RTIs) in patients with AGS due to mutations in TREX1, components of the RNase H2 complex or SAMHD1, using combined abacavir, zidovudine, and lamivudine for 12 months [6]. A reduction of interferon antiviral activity in blood and cerebrospinal fluid (CSF), a fall in interferon alpha protein levels in serum, and a decrease in the expression of genes induced by interferon in blood were observed under treatment, without obvious clinical efficacy. More recently, the description of a promising effect of JAK1/2 inhibitors in AGS, used with the aim of blocking JAK1 at the type I interferon receptor, suggests that this class of drug represents an important approach to the treatment of this devastating disease [7, 8]. In 2020, Vanderver et al reported an open-label study of 35 patients with AGS treated with baricitinib over a period of 12 to 44 months [8]. Skin and systemic manifestations of the disease responded apparently favorably to JAK1/2 inhibition, as has been described by others [9–12]. However, the benefit of such therapy on the neurological component of AGS was less clear, with limited developmental gains seen. In this regard, we recently described one child with RNASEH2B-related AGS in whom we observed the appearance of disease features at age 15 months, despite the use of ruxolitinib starting at 5 months of age when the child was apparently clinically asymptomatic [13].

Here, in a real-world approach study, we report our off-label experience of the use of JAK1/2 inhibition in 11 patients with AGS, with a median follow-up of 17 months (range 12 to 48 months) providing extensive clinical (AGS neurologic severity scale [8, 14]; polyhandicap severity scale [15]; dystonia rating [16–18]; caregiver assessment of activities of daily living [19]), radiological, and biological data.

## Methods

### Off-Label Treatment Study

We conducted a real-word approach study in AGS with off-label treatment with a JAK1/2 inhibitor, either ruxolitinib or baricitinib, from September 2017 to September 2022. Treatment was initiated with parental written consent.

### Inclusion Criteria

All molecularly confirmed cases of AGS known to us were assessed for treatment, involving an extended clinical evaluation conducted by two expert clinicians in pediatric immunology and neurology. Patients selected for treatment were followed up as detailed in the next section.

### Design of the Real-Word Approach Study for Treated Patients

Clinical and neurological assessments were performed at each visit (month (M) 0, M3, M6, M9, M12, and then every 6 months). All patients were assessed by a single neuro-pediatrician (Marie Hully, MH). Specific scales were used to record developmental status and evolution, i.e., the AGS neurologic severity scale developed by Adang et al [14] and the polyhandicap severity scale developed by Rousseau et al [15]. Where present, dystonia and other movement disorders were assessed using the Barry Albright Dystonia Scale [16] (BADS) and the Movement Disorder Childhood Rating scales [17, 18] (MDCS). Parents were asked to complete the Caregiver questionnaire [19], so as to capture their perception of their child’s status in everyday life.

Cerebral magnetic resonance imaging (MRI) was performed at drug initiation and annually thereafter. MRI data for each patient were systematically reviewed by an expert neuroradiologist (Nathalie Boddaert, NB). Cerebral atrophy was assessed according to ventricular dilatation and periventricular spaces on the same sequence (T2-weighted images) at two given follow-up times.

Routine laboratory examinations and screening for infections (BK virus, CMV, EBV by PCR in blood) were performed at each visit. We recorded interferon scores by measuring the expression of a panel of interferon-stimulated genes (ISGs) in whole blood [20] by RT-qPCR [20] or Nanostring technology as in [21], before treatment initiation and every 3 months thereafter. Interferon alpha protein levels in serum and CSF were measured using a digital ELISA [22]. Neopterin levels in serum and CSF were measured using liquid chromatography tandem mass spectrometry in the multiple reaction monitoring mode (LC-MSMS), and reference values used as reported [23]. CSF examination was performed at enrolment, M3, M6, and then bi-annually. Blood and CSF were taken for pharmacokinetic (PK) measurements of ruxolitinib and baricitinib using LC-MSMS analytical validated methods where possible.

### Patients Assessed and Not Treated

During the study period, 12 AGS patients were reviewed and, after extended clinical evaluation (see Tables S2 and S3), not started on a JAK inhibitor. The reasons for non-inclusion were CNS-restricted involvement with already-fixed severe brain damage and an absence of CSF inflammation where assessed (*n* = 7), the presence of cardiomyopathy (*n* = 1), minimal skin vasculopathy with no neurological involvement (*n* = 1), and parental refusal (*n* = 3). Assessment of AGS developmental scale at screening is displayed in Figure S7. Longitudinal natural history data were available for 8 of 12 of these patients (Table S2 and Figure S7).

### Statistical Analysis

Analyses were performed with PRISM software (v6 for Macintosh, GraphPad Inc.) as indicated (paired *t* test or Kruskal-Wallis test). A *p* value less than 0.05 was considered significant.

## Results

A total of 11 patients with a molecularly proven diagnosis of AGS (*RNASEH2B*, *n* = 4; *ADAR1*, *n* = 2; *TREX1*, *n* = 2; *IFIH1*, *n* = 2; *RNU7-1*, *n* = 1) were treated with a JAK1/2 inhibitor for a median period of 17 (range, 12–48) months (Table 1). Median age at onset of disease was 4 months (range antenatal, 17 months), with presentation typically encompassing the core clinical features of AGS (acute encephalitis followed by regression and loss of developmental milestones). Bilateral striatal necrosis was noted in 1 patient (P6) with ADAR1-related disease. As previously described, 1 patient (P5) was treated prior to the onset of clinical disease features [13]. Four patients demonstrated significant extra-neurological involvement, specifically cutaneous lesions (chilblains (*n* = 2), psoriatic lesions (*n* = 1), erythermalgia (*n* = 1)), autoimmune cytopenias (*n* = 1), and glaucoma (*n* = 2). Median age at drug initiation was 1.7 (range, 0.38 to 16.6) years, with the main indications for treatment being systemic features (*n* = 4) (Figure S1), episodic neurological regression (*n* = 5), discomfort (*n* = 1), and pre-emptive treatment (*n* = 1) [13] (Table 2). Of note, treatment was initiated early in the course of the disease in 2 patients (P7 and P8) with the hope of minimizing further neurological progression. The first 5 patients treated received the JAK1/2 inhibitor ruxolitinib at a median dosage of 15 mg/m^2^/day, subsequently increased to a median dose of 30 mg/m^2^/day at last follow-up. Following the report of Vanderver et al [8], 6 further patients were treated with the JAK1/2 inhibitor baricitinib, employing dosages similar to the ones used by Vanderver and colleagues [8]. Only P2 received concomitant immunosuppressive treatment (i.e., oral steroids) at the time of initiation of JAK inhibition.

**Table 1:** Clinical characteristics of treated patients

	AGS number	Genotype	Mutation	Sex	Age at onset	Age at diagnosis	CNS features	Skin features	Autoimmunity	Other
P1	AGS0769	*TREX1*	p.Ala114His/p.(Pro212Hisfs*65)	M	3 m	14 y	Developmental delay, progressive spastic and dystonic tetraparesis	Chilblains	None	Glaucoma, ICC
P2	AGS1537	*ADAR1*	p.Gly1007Arg Hz	F	17 m	10 y	Spastic-dystonic tetraparesis	Erythermalgia (12 y)	AIHA and ITP (10 y)	Glaucoma, ICC
P3	AGS2180	*IFIH1*	p.Arg779Cys Hz	F	6–12 m	2.5 y	Progressive spastic diplegia with white matter disease and brain atrophy on MRI	None	None	
P4	AGS2437.1	*RNASEH2B*	p.Ala177Thr/IVS6-13G>A	M	2 m	10 m	Developmental delay, dystonic-spastic tetraparesis	None	None	
P5	AGS2437.2	*RNASEH2B*	p.Ala177Thr/IVS6-13G>A	M	15 m	Neonatal	Normal until 14 m; then,spastic-dystonic tetraparesis, developmental delay	None	None	
P6	AGS2350	*ADAR1*	p.Pro193Ala, p.Gly998Glu	M	8 m	3.4 y	Striatal necrosis, progressive generalized dystonia	None	None	
P7	AGS2931	*RNASEH2B*	p.Ala177Thr Ho	M	1 m	2 m	Poor eye contact, axial hypotonia, spastic-dystonic tetraparesis, epilepsy	None	None	Poor feeding
P8	AGS2895	*RNU7-1*	n.28C>g, n.40_47del	M	4 m	12 m	Nystagmus, microcephaly, dystonia	None	None	None
P9	AGS3031	*RNASEH2B*	p.Ala177Thr Ho	F	8 m	17 m	Spastic-dystonic tetraparesis, axial hypotonia	None	None	Poor feeding
P10	AGS3065	*IFIH1*	p.Arg779Cys Hz	M	3 m	5 m	Spastic-dystonic tetraparesis	Severe dermatitis	None	Poor feeding
P11	AGS2831	*TREX1*	p.Cys208Leufs33* Ho	M	Antenatal	Antenatal	Severe encephalopathy, spastic-dystonic tetraparesis, poor interaction	Chilblains (onset at 6 m)	None	ICC

Abbreviations: *AIHA* autoimmune hemolytic anemia, *F* female, *Ho* homozygous, *Hz* heterozygous, *ICC* intracranial calcification, *ITP* immune thrombocytopenia, *M* male, *m* months, *MRI* magnetic resonance imaging, *y* years

**Table 2:** Summary of JAK inhibitor treatment in the cohort

	AGS number	Genotype	Treatment indication	Age at initiation (y)	Follow-up (m)	JAKi	Initial/max dosing (mg/m^2^/day or mg/kg/day*)	Dosing at last follow-up (mg/m^2^/day or mg/kg/day*)	Concomitant treatments at JAKi initiation (time of discontinuation)	Infections under JAKi	Other adverse side effects
P1	AGS0769	*TREX1*	Skin	16.6	40	Ruxolitinib	15/30	15	None	SARS-Cov-2^#^	Weight gain
P2	AGS1537	*ADAR1*	AIHA and ITP, enteral feeding	12.7	48	Ruxolitinib	20/30	30	Steroids (M6)	None	Weight gain, anemia
P3	AGS2180	*IFIH1*	Neuro	3.7	45	Ruxolitinib	20/30	30	None	SARS-Cov-2^§^	
P4	AGS2437.1	*RNASEH2B*	Neuro	1.9	24	Ruxolitinib	12/30	30	None	SARS-Cov-2^§^	
P5	AGS2437.2	*RNASEH2B*	Pre-emptive	0.42	24	Ruxolitinib	13/30	30	None	SARS-Cov-2^§^	Weight gain
P6	AGS2350	*ADAR1*	Neuro	4.41	16	Baricitinib	0.37/0.5*	0.5*	None	None	
P7	AGS2931	*RNASEH2B*	Discomfort	0.38	17	Baricitinib	0.8*	0.5*	None	None	
P8	AGS2895	*RNU7*	Neuro	0.92	16	Baricitinib	0.7*	0.6*	None	Osteomyelitis	
P9	AGS3031	*RNASEH2B*	Neuro, poor feeding (NG tube)	1.67	12	Baricitinib	0.6*	0.6*	None	Pneumococcal septicemia**; SARS-Cov-2^#^	Anemia
P10	AGS3065	*IFIH1*	Severe dermatitis, poor feeding (NG tube), discomfort	0.42	16	Baricitinib	0.6*	0.6*	None	Pneumococcal meningitis**	
P11	AGS2831	*TREX1*	Skin	1.42	14	Baricitinib	0.7*	0.7*	None	Aspiration pneumonia, pyelonephritis	Anemia, thrombocytopenia

Abbreviations: *AIHA* autoimmune hemolytic anemia, *JAKi* JAK inhibitors, *ITP* immune thrombocytopenia, *M* month of follow-up, *m* months, *Neuro* episodic neurological regression, *NG* nasogastric, *y* year

*Dosing in mg/kg/day

^#^SARS-Cov-2 infection restricted to upper respiratory tract involvement

^§^Asymptomatic SARS-Cov-2 infection

**Non-vaccine serotype *Streptococcus pneumonia*

### Side Effects

Side effects were recorded in 7 patients (Table 2). Cytopenia was documented in 3 patients: P2 and P9 developed anemia (with a nadir of hemoglobin at 8 and 9 g/dl respectively), while anemia and thrombocytopenia were noted in P11. Four patients, all under baricitinib, experienced bacterial infections necessitating 5 hospitalizations (osteomyelitis; pneumococcal septicemia; pneumococcal meningitis; aspiration pneumonia; pyelonephritis). Of note, the 2 *Streptococcus pneumoniae* infections occurred in children fully vaccinated with pneumococcal conjugated vaccine (PCV13) and were due to non-vaccinal strains (17F and 24F). SARS-CoV-2 infection was documented in 5 patients, and was asymptomatic (*n* = 3) or restricted to upper respiratory tract symptoms (*n* = 2). No serious viral infection was observed in the cohort, and no BK viremia was documented. Excessive weight gain while on treatment with ruxolitinib was noted in 3 patients.

### Effect on Extra-Neurological Disease Features

We observed a significant improvement of chilblain skin lesions, with a complete remission in P1 during the summer months and the recurrence of mild, non-painful lesions in the winter, and a partial response in P11 (Figure S1A). A complete and durable remission of psoriatic-like lesions was seen in P10 (Figure S1B). In P2, remission of significant autoimmune cytopenia was achieved, allowing tapering and cessation of steroids at M6, with prolonged remission up to last follow-up at M48, in contrast to steroid dependence before initiation of JAK inhibition. Erythermalgia in P2 was also rapidly controlled. In addition, treatment was associated with a complete weaning off of enteral feeding in 3 of 4 patients, allowing removal of a gastrostomy in P2. Parents reported a reduction in fatigue and an improvement in the general condition of their children, with a gain in comfort where irritability was present prior to initiation of JAK inhibition (see below).

### Effect on Neurological Disease Features

**Fig. 1:**
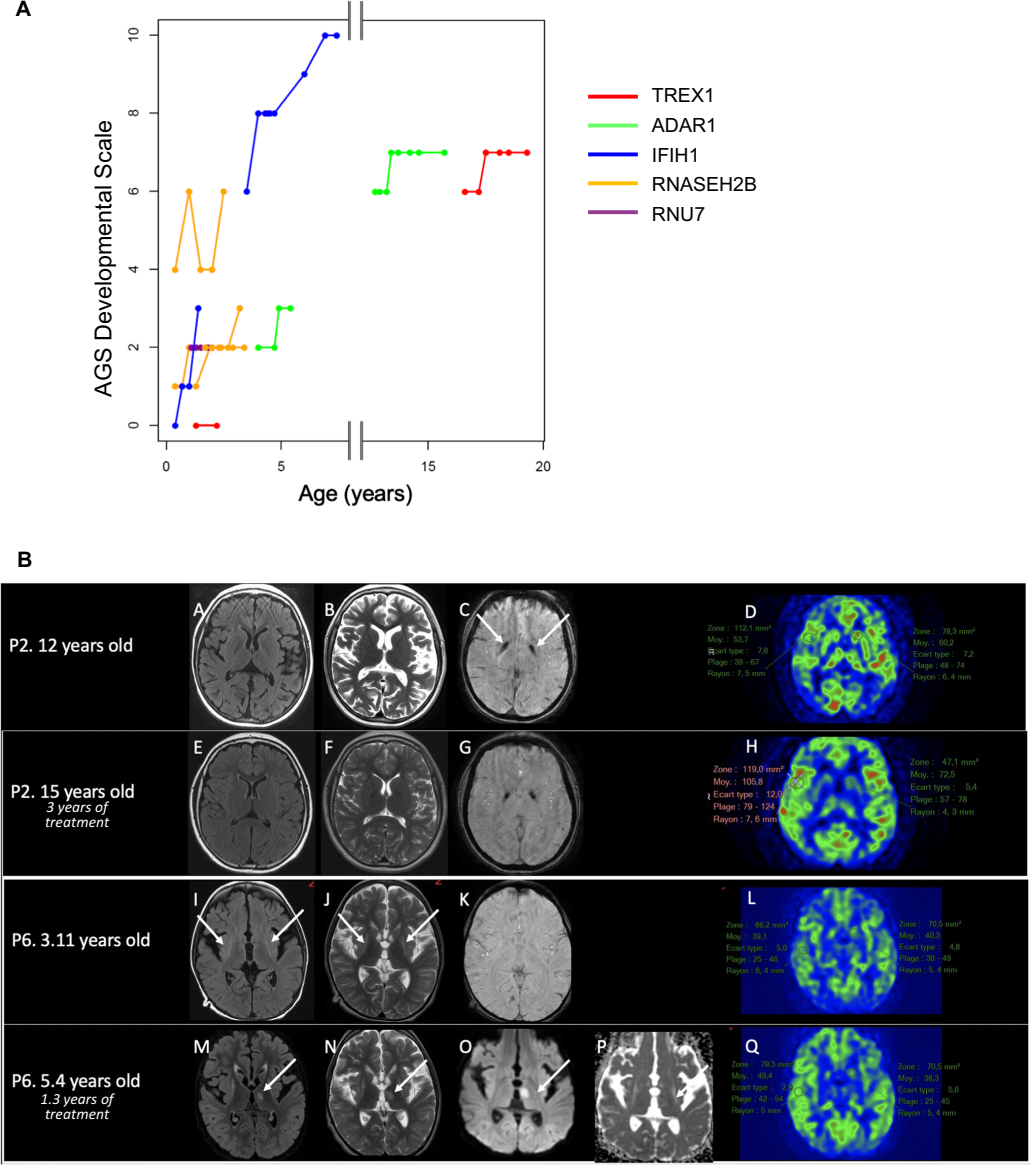
Clinical and radiological response to treatment. **A** Evolution of the AGS developmental scale [14] in patients under JAK inhibition according to age and mutant genotype. Overall, a slight (mean, 1.2 points) but significant (*p* = 0.036, Kruskal-Wallis test) improvement on AGS severity scale was observed. **B** Brain magnetic resonance imaging (MRI) in 2 affected patients before and under treatment. The first patient (P2), 12 years old at treatment initiation, demonstrated global supra-tentorial atrophy on axial FLAIR (subpanel A) and T2 (subpanel B)–weighted images associated with calcifications of the globi pallidi on T2 star–weighted images (subpanel C, arrows). After 3 years of treatment, at age 15 years, atrophy had decreased (axial FLAIR (subpanel E), axial T2–weighted images (subpanel F)). On arterial spin labeling sequence (ASL) (subpanels D, H), cortical cerebral blood flow (CBF) increased between the ages of 12 and 15 years (CBF was measured at 53 ml/min/100 g per tissue in the temporal cortex at 12 years (subpanel D), increasing to 105 ml/min/100 g per tissue at 15 years old (subpanel H)). P6 demonstrated bilateral striatal anomalies (arrows) on axial FLAIR (subpanel I) and on axial T2–weighted images (subpanel K) at age 3.11 years. After 1.6 years of treatment, the appearance on the axial FLAIR–weighted (subpanel M) and axial T2–weighted (subpanel N) imaging showed nodular hyperintensity in the left thalamus (arrow). The diffusion-weighted images (subpanel O) showed an increased diffusion coefficient (subpanel P). On ASL (subpanels L, Q), CBF in the basal ganglia and cortex (about 40 ml/min/100 g tissue) (subpanels L and Q respectively) remained unchanged between the 2 periods

Neurological scales were assessed up to a median of 1.4 (range, 0.9–3.2) years after drug initiation. On the AGS neurologic severity scale, we observed a statistically significant, but minor, improvement at M9, M12, M18, and M24 (*p* = 0.018, 0.010, 0.012, and 0.016 respectively; paired *t* test), as compared to M0 (Fig. 1A), with an overall global significant improvement (mean 1.2 points) over time (*p* = 0.036, Kruskal-Wallis test). The greatest improvements recorded concerned the ability to smile and vocalize (not significant, *p* = 0.17, and *p* = 0.038, respectively; paired *t* test) (Figure S2A). No statistically significant difference between M0 and different assessments was observed for movement disorders on either the BADS or MDCS scale (not shown), on the polyhandicap severity scale (Figure S2B) or on the caregiver assessment scale beyond an improvement in comfort recorded at 9 months (*p* = 0.032, paired *t* test) (Figure S2C). Of importance, we observed onset of neurological disease in one patient under treatment [13].

Assessment of brain MRI and cerebral blood flow (CBF) by arterial spin labeling (ASL) was possible at screening and during follow-up in 10 patients (P1–P10). We observed no change in brain imaging in P1 (not shown), while atrophy decreased and ASL increased in P2 who was on steroids at the initiation of ruxolitinib (Fig. 1B). Atrophy also improved in five further patients (P3, P4, P5, P7, P8), all of whom started treatment before the age of 4 years, while CBF remained unchanged. In the 2 other patients undergoing serial MRI examination (P9 and P10), we observed no change in atrophy but a slight improvement in white matter signal in P9 (possibly accounted for by brain maturation), whereas atrophy and white matter disease worsened in P10.

### Disease Course of P6 Under JAK Inhibition

The clinical and radiological follow-up of ADAR1-mutated patient P6 deserves a special note. Despite complete normalization of the interferon score in blood (Figure S3), and optimal therapeutic dosing with baricitinib, at M9, he experienced increased discomfort and hypertonia. Baricitinib was recordable in the CSF, being around 14% of the drug concentration in serum. No systemic features, including fever, were observed. Brain MRI at M12 revealed new inflammatory lesions (Fig. 1B) highly evocative of a disease flare. Systemic and central nervous system (CNS) infections were ruled out, with no pathogen detected using next-generation sequencing (NGS). Subsequent brain MRI 3 months later, at M15, showed an evolution toward cavitation. Altogether, these features suggest a flare of disease uncontrolled by optimal dosing with baricitinib.

### Effect on Biomarkers

**Fig. 2:**
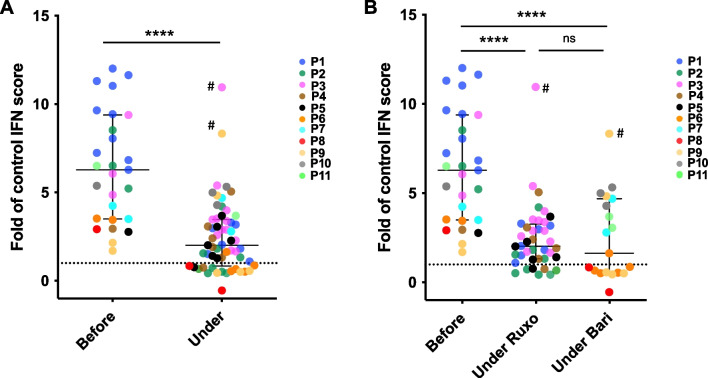
Response of type I interferon biomarkers to treatment. Interferon scores assessed by qRT-PCR [20] or Nanostring [21] in the whole blood of patients (P1–P11) before and under treatment with a JAK inhibitor, either as composite data (**A**) or according to the JAK inhibitor used, i.e., ruxolitinib (Ruxo) or baricitinib (Bari) (**B**). Data are expressed as fold values of the normal interferon score (i.e., 2.466 and 2.724 for qRT-PCR and Nanostring Technologies respectively). Horizontal bars indicate median values and interquartile ranges. Comparison between the two groups was performed using Mann-Whitney test (****p < 0.0001). # indicates two samples taken during SARS-CoV-2 infection

Lumbar puncture was performed at screening in 10 of 11 patients, with an inflammatory phenotype (elevated number of white blood cells and protein level in the CSF) observed in 5 individuals (Table S1). These CSF features normalized under treatment in 4 patients (Table S1). Interferon scores were positive before treatment in all eleven patients (Fig. 2A), and interferon alpha protein levels were elevated in the CSF and the blood of all 9 patients assessed at screening (Table S1). There was a significant reduction of the median interferon score under treatment, with a normalization of the interferon signature in 6 patients (Fig. 2A and Figure S4), whereas interferon alpha protein concentration remained elevated in the CSF (albeit with intraindividual variability and a decreasing trend in some patients, Figure S5). Of note, we observed a comparable reduction in the median interferon score with either JAK inhibitor (Fig. 2B). We also monitored CSF neopterin levels in 8 of 11 patients and observed a trend toward decreased values under treatment (Figure S6).

### PK Data

Ruxolitinib concentration was measured in the serum and the CSF of all 5 patients treated [13]. Baricitinib concentration was assessed in 5 of the 6 patients receiving this drug. Results of the area under the curve (AUC) at 0–24 h for baricitinib were higher than the PK data reported by Kim et al [24] (median of 1332 ng.h/ml versus 562 ng.h/ml, respectively; Table S4), despite using the dosing recommended in that study (and in Vanderver et al [8]). In addition, baricitinib was measured in the CSF of 5 patients (11 CSF samples), with a median value of 7.26 ng/ml (range, 1.24–15.25), and individual levels seemingly dependent upon the time from last drug intake (Table S5). Of note, drug concentrations of baricitinib in the CSF were only 10–20% of those recorded in the blood, which is consistent with our previously published data [13], where the concentration of ruxolitinib in the CSF was only around 10% of that measured in the serum.

## Discussion

Summarizing our data, in a study of 11 patients with AGS treated for a median period of 17 months, while we observed a good response of systemic disease features to JAK1/2 inhibition, we saw only limited or no quantifiable benefit on the neurologic manifestations of the disorder.

As previously documented, the characteristic skin lesions seen in AGS frequently respond well to JAK1/2 inhibition, as has been described in other type I interferonopathies [25–27]. Furthermore, we observed sustained clinical and biological remission of an established steroid-dependant hemolytic anemia in one patient, even if JAK1/2 inhibition is not considered as a conventional treatment of autoimmune cytopenias. Thus, beyond their use in monogenic inborn errors of the JAK-STAT pathway [28], the benefit of JAK inhibition in the treatment of autoimmune cytopenias seen in the context of interferon-related diseases, such as systemic lupus erythematosus, deserves consideration. Notably, treatment seemed to facilitate weaning off of enteral feeding, which could be related to the relief of an anorectic effect of the type I interferon pathway, or decreased leptin signaling under JAK2 inhibition [29]. In line with this, 3 patients demonstrated excessive weight gain on treatment.

Using the AGS neurologic severity scale developed by Adang et al [14], our results were in line with those reported by Vanderver et al [8], with a median gain of 1 point on the 11-point scale, and improvement mainly confined to social smile and vocalization, the latter consistent with the description of caregivers reporting their children to be more comfortable (this improvement was statistically significant at M9 compared to M0). However, there was no change in other scales assessing either the severity of multiple disabilities (polyhandicap [15]), movement disorders [16–18], or other domains of everyday-life care [19] (assessed by parents).

Interpretation of brain imaging data collected over the study period reported here is unclear. An important improvement of brain volume, and increased cerebral blood flow, was observed in P2 after 1 year of treatment, but baseline evaluation was performed while the patient was taking corticosteroids. Improvement of brain atrophy in 5 further patients might represent an effect of treatment, but may also relate to the natural history of the disease in some cases [30].

The positive effects of JAK1/2 inhibition observed on the systemic features of AGS suggest that these drugs address a biological process relevant to disease pathogenesis. Further, we observed a significant reduction of interferon scores under treatment. However, while appreciating that any improvement in neurological status is of importance, we consider it now evident that JAK1/2 inhibition does not afford major benefits in terms of neurological disease in the majority of patients with AGS so far treated. We consider two factors to likely explain this finding. Firstly, we note the late stage in the disease process at which treatment is initiated in most patients. Thus, while in the series reported here and that of Vanderver et al [8] the median age at symptom onset was 4–6 months, the median age at the initiation of JAK inhibition was 1.7 and 2.9 years in the two studies respectively. Beyond this point, we draw attention to a second important observation, the fact that 2 patients in our cohort demonstrated neurological progression while on treatment. Thus, new inflammatory lesions were observed in the white matter of P6 (Fig. 1B) despite complete control of interferon signaling in the periphery (Figure S3) and optimal dosage of baricitinib as assessed by PK. Furthermore, as already reported, we observed the onset of AGS in P5 at age 15 months, despite pre-emptive treatment with ruxolitinib starting at age 5 months [13]. We consider inadequate CNS drug penetration to be the most probable explanation for such treatment failure. Evidence supporting this statement comes from the fact that in the case of P5, the concentration of ruxolitinib in the CSF was consistently found to be only 10% of that in blood [13], while in the current study, levels of baricitinib in the CSF were 10–20% of those recorded in blood. Aligned with this, we draw attention to a marked, and persistent, elevation of interferon alpha protein in the CSF (Figure S5), contrasting with a considerable reduction of interferon signaling in blood.

The safety profile related to JAK1/2 inhibition is both a short- and long-term concern. Vanderver et al reported the death of 2 patients while on treatment with baricitinib [8], including one attributed to pulmonary hypertension, a feature described in AGS [31] and that might be exacerbated by JAK inhibition [32]. The second patient, demonstrating a severe manifestation of AGS with multisystem involvement, was found to have a fungal pneumonia at autopsy that was considered possibly attributable to study medication. Cytopenias occurred in 3 patients of our cohort, which we judge to be beyond the hematological abnormalities seen in patients with untreated AGS [33]. Notably, bacterial infections occurred in 4 patients in our cohort, including severe pneumococcal infections in 2. Both of these patients, each aged less than 2 years, had received pneumococcal conjugated vaccine (PCV13) and not pneumococcal polysaccharide vaccine (PPSV23). Susceptibility to encapsulated bacteria has not been reported in AGS, or in patients on JAK1/2 inhibitors used to treat other conditions. However, experience of the long-term exposure to high doses of JAK1/2 inhibitors starting in infancy is still limited, and our observation indicates the need for vigilance in this regard, with careful monitoring of dosage [24]. This observation also supports the importance of vaccination of patients with AGS prior to initiation of JAK inhibition. Interestingly, functional asplenia and poor polysaccharide antibody responses have been described in the context of SAVI (STING-associated vasculopathy with onset in infancy) [34], another type I interferonopathy, and further investigations are warranted to delineate the underlying mechanism. Finally, although we did not observe such complications in this cohort, concerns about cytokine crisis following overly prompt withdrawal of JAK1/2 inhibition are of very high importance [27, 35].

Beyond the issues described above, AGS presents particular difficulties in clinical trial design. Thus, while AGS is a severe disease, with 19% of patients reported in the largest natural history study published to date [2] having died, and 74% being profoundly disabled, the rarity of the disorder and a well-recognized, sometimes extreme, variation in disease expression between siblings represent major challenges in the assessment of treatment efficacy. We also draw attention to the fact that some genotypes may represent better responder groups, in particular *SAMHD1*, *ADAR1*, and *IFIH1*, in which the disease course may be less severe [36–38], and that age is a confounding factor for milder phenotypes where patients may still acquire developmental milestones irrespective of treatment. Therefore, it seems difficult to employ the same scale to evaluate differentially affected patients, with Goal Attainment Scaling (GAS) [39] representing an alternative, or combined, assessment tool in future AGS-related clinical trials.

As a further point, it is already known that markers of interferon status and CNS inflammation can fall in the months beyond the initial encephalopathic period [2, 20, 40], so that assessing changes in biomarkers needs to take this possibility into account. In addition, we draw attention to the remarkable observation in P5 of grossly elevated levels of interferon alpha protein (14,989 fg/ml: normal < 10 fg/ml) in the CSF at the age of 4 months, 11 months prior to the onset of clinical neurological disease [13]. Consistent with previously reported data [41], neopterin levels showed a tendency to decrease under JAK1/2 inhibition. There is still a need for biomarkers that accurately reflect brain disease and allow therapeutic monitoring. To this end, CSF neopterin levels [41] and markers of brain damage [42] might represent relevant indices.

Overall, our report indicates a benefit of JAK1/2 inhibition on certain systemic features of AGS, but a minimal measurable effect on the associated neurological phenotype. Such benefits need to be considered against the risks of treatment with these drugs over the long term. All told, we conclude that there remains a clear need for other approaches to the treatment of this devastating condition [43]. Early diagnosis and adequate CNS penetration likely remain the major factors determining the efficacy of therapy in preventing irreversible brain damage, implying the potential importance of early and rapid genetic testing and the consideration of intrathecal drug delivery.

## Supplementary Information

ESM 1 (PPTX 19293 kb)

ESM 2 (DOCX 68.7 kb)

## Declarations

### Provenance and Peer Review

The patients and public were not involved in clinical research or co-production of research.

## References

[1] Crow YJ, Stetson DB. The type I interferonopathies: 10 years on. Nat Rev Immunol. 2021; 10.1038/s41577-021-00633-9 PMC8527296 34671122

[2] Crow YJ, Chase DS, Lowenstein Schmidt J, Szynkiewicz M, Forte GMA, Gornall HL (2015). Characterization of human disease phenotypes associated with mutations in TREX1, RNASEH2A, RNASEH2B, RNASEH2C, SAMHD1, ADAR, and IFIH1. Am J Med Genet A.

[3] Livingston JH, Lin J-P, Dale RC, Gill D, Brogan P, Munnich A (2014). A type I interferon signature identifies bilateral striatal necrosis due to mutations in ADAR1. J Med Genet.

[4] D’Arrigo S, Riva D, Bulgheroni S, Chiapparini L, Lebon P, Rice G (2008). Aicardi-Goutières syndrome: description of a late onset case. Dev Med Child Neurol.

[5] Volkman HE, Stetson DB (2014). The enemy within: endogenous retroelements and autoimmune disease. Nat Immunol.

[6] Rice GI, Meyzer C, Bouazza N, Hully M, Boddaert N, Semeraro M (2018). Reverse-Transcriptase Inhibitors in the Aicardi–Goutières Syndrome. N Engl J Med.

[7] Kothur K, Bandodkar S, Chu S, Wienholt L, Johnson A, Barclay P (2018). An open-label trial of JAK 1/2 blockade in progressive IFIH1-associated neuroinflammation. Neurology.

[8] Vanderver A, Adang L, Gavazzi F, McDonald K, Helman G, Frank DB (2020). Janus Kinase Inhibition in the Aicardi-Goutières Syndrome. N Engl J Med.

[9] McLellan KE, Martin N, Davidson JE, Cordeiro N, Oates BD, Neven B (2018). JAK 1/2 Blockade in MDA5 Gain-of-Function. J Clin Immunol.

[10] Briand C, Frémond M-L, Bessis D, Carbasse A, Rice GI, Bondet V (2019). Efficacy of JAK1/2 inhibition in the treatment of chilblain lupus due to TREX1 deficiency. Ann Rheum Dis.

[11] Zheng S, Lee PY, Wang J, Wang S, Huang Q, Huang Y (2020). Interstitial Lung Disease and Psoriasis in a Child With Aicardi-Goutières Syndrome. Front Immunol.

[12] Broser P, von Mengershausen U, Heldt K, Bartholdi D, Braun D, Wolf C (2022). Precision treatment of Singleton Merten syndrome with ruxolitinib: a case report. Pediatr Rheumatol Online J.

[13] Neven B, Al Adba B, Hully M, Desguerre I, Pressiat C, Boddaert N (2020). JAK Inhibition in the Aicardi-Goutières Syndrome. N Engl J Med.

[14] Adang LA, Gavazzi F, Jawad AF, Cusack SV, Kopin K, Peer K (2020). Development of a neurologic severity scale for Aicardi Goutières Syndrome. Mol Genet Metab.

[15] Rousseau MC, Baumstarck K, Hamouda I, Valkov M, Felce A, Khaldi-Cherif S (2021). Development and initial validation of the polyhandicap severity scale. Rev Neurol.

[16] Barry MJ, VanSwearingen JM, Albright AL (1999). Reliability and responsiveness of the Barry-Albright Dystonia Scale. Dev Med Child Neurol.

[17] Battini R, Sgandurra G, Petacchi E, Guzzetta A, Di Pietro R, Giannini MT (2008). Movement disorder-childhood rating scale: reliability and validity. Pediatr Neurol.

[18] Battini R, Guzzetta A, Sgandurra G, Di Pietro R, Petacchi E, Mercuri E (2009). Scale for evaluation of movement disorders in the first three years of life. Pediatr Neurol.

[19] Schneider JW, Gurucharri LM, Gutierrez AL, Gaebler-Spira DJ (2001). Health-related quality of life and functional outcome measures for children with cerebral palsy. Dev Med Child Neurol.

[20] Rice GI, Forte GMA, Szynkiewicz M, Chase DS, Aeby A, Abdel-Hamid MS (2013). Assessment of interferon-related biomarkers in Aicardi-Goutières syndrome associated with mutations in TREX1, RNASEH2A, RNASEH2B, RNASEH2C, SAMHD1, and ADAR: a case-control study. Lancet Neurol.

[21] Lepelley A, Martin-Niclós MJ, Le Bihan M, Marsh JA, Uggenti C, Rice GI, et al. Mutations in COPA lead to abnormal trafficking of STING to the Golgi and interferon signaling. J Exp Med. 2020;217 10.1084/jem.20200600 PMC7596811 32725128

[22] Rodero MP, Decalf J, Bondet V, Hunt D, Rice GI, Werneke S (2017). Detection of interferon alpha protein reveals differential levels and cellular sources in disease. J Exp Med.

[23] Ormazabal A, García-Cazorla A, Fernández Y, Fernández-Alvarez E, Campistol J, Artuch R (2005). HPLC with electrochemical and fluorescence detection procedures for the diagnosis of inborn errors of biogenic amines and pterins. J Neurosci Methods.

[24] Kim H, Brooks KM, Tang CC, Wakim P, Blake M, Brooks SR (2018). Pharmacokinetics, pharmacodynamics, and proposed dosing of the oral JAK1 and JAK2 inhibitor baricitinib in pediatric and young adult CANDLE and SAVI patients. Clin Pharmacol Ther.

[25] Frémond M-L, Rodero MP, Jeremiah N, Belot A, Jeziorski E, Duffy D (2016). Efficacy of the Janus kinase 1/2 inhibitor ruxolitinib in the treatment of vasculopathy associated with TMEM173-activating mutations in 3 children. J Allergy Clin Immunol.

[26] Sanchez GAM, Reinhardt A, Ramsey S, Wittkowski H, Hashkes PJ, Berkun Y (2018). JAK1/2 inhibition with baricitinib in the treatment of autoinflammatory interferonopathies. J Clin Invest.

[27] Frémond M-L, Hadchouel A, Berteloot L, Melki I, Bresson V, Barnabei L (2021). Overview of STING-Associated Vasculopathy with Onset in Infancy (SAVI) Among 21 Patients. J Allergy Clin Immunol Pract.

[28] Hadjadj J, Frémond M-L, Neven B (2021). Emerging place of JAK inhibitors in the treatment of inborn errors of immunity. Front Immunol.

[29] Mollé N, Krichevsky S, Kermani P, Silver RT, Ritchie E, Scandura JM (2020). Ruxolitinib can cause weight gain by blocking leptin signaling in the brain via JAK2/STAT3. Blood.

[30] Tonduti D, Izzo G, D’Arrigo S, Riva D, Moroni I, Zorzi G (2019). Spontaneous MRI improvement and absence of cerebral calcification in Aicardi-Goutières syndrome: Diagnostic and disease-monitoring implications. Mol Genet Metab.

[31] Adang LA, Frank DB, Gilani A, Takanohashi A, Ulrick N, Collins A (2018). Aicardi goutières syndrome is associated with pulmonary hypertension. Mol Genet Metab.

[32] Low AT, Howard L, Harrison C, RMR T (2015). Pulmonary arterial hypertension exacerbated by ruxolitinib. Haematologica.

[33] Adang LA, Gavazzi F, D’Aiello R, Isaacs D, Bronner N, Arici ZS, et al. Hematologic abnormalities in Aicardi Goutières Syndrome. Mol Genet Metab. 2022; 10.1016/j.ymgme.2022.06.003 PMC9357135 35786528

[34] Bijker EM, Rösler B, Hoppenreijs E, Henriet S, van der Flier M (2021). Functional Asplenia and Specific Polysaccharide Antibody Deficiency in a Girl with SAVI. J Clin Immunol.

[35] Tefferi A, Pardanani A (2011). Serious adverse events during ruxolitinib treatment discontinuation in patients with myelofibrosis. Mayo Clin Proc.

[36] Adang L, Gavazzi F, De Simone M, Fazzi E, Galli J, Koh J (2020). Developmental outcomes of aicardi goutières syndrome. J Child Neurol.

[37] Rice GI, Kitabayashi N, Barth M, Briggs TA, Burton ACE, Carpanelli ML (2017). Genetic, Phenotypic, and Interferon Biomarker Status in ADAR1-Related Neurological Disease. Neuropediatrics.

[38] Rice GI, Park S, Gavazzi F, Adang LA, Ayuk LA, Van Eyck L (2020). Genetic and phenotypic spectrum associated with IFIH1 gain-of-function. Hum Mutat.

[39] Steenbeek D, Ketelaar M, Galama K, Gorter JW (2007). Goal attainment scaling in paediatric rehabilitation: a critical review of the literature. Dev Med Child Neurol.

[40] Lodi L, Melki I, Bondet V, Seabra L, Rice GI, Carter E (2021). Differential Expression of Interferon-Alpha Protein Provides Clues to Tissue Specificity Across Type I Interferonopathies. J Clin Immunol.

[41] Han VX, Mohammad SS, Jones HF, Bandodkar S, Crow YJ, Dale RC (2022). Cerebrospinal fluid neopterin as a biomarker of treatment response to Janus kinase inhibition in Aicardi-Goutières syndrome. Dev Med Child Neurol.

[42] Izzotti A, Fazzi E, Orcesi S, Cartiglia C, Longobardi M, Capra V (2008). Brain damage as detected by cDNA-microarray in the spinal fluid of patients with Aicardi-Goutieres syndrome. Neurology.

[43] Crow YJ, Neven B, Frémond M-L (2021). JAK inhibition in the type I interferonopathies. J Allergy Clin Immunol.

