# Supplementary material for: JAK Inhibition in Aicardi-Goutières Syndrome: a Monocentric Multidisciplinary Real-World Approach Study
Source: J Clin Immunol. 2023 May 12;43(6):1436–47. doi: 10.1007/s10875-023-01500-z (PMC10175907; doi:10.1007/s10875-023-01500-z)
Supplement: Supplementary file 1 — (PPTX 19293 kb) [file 10875_2023_1500_MOESM1_ESM.pptx]

## Slide 1
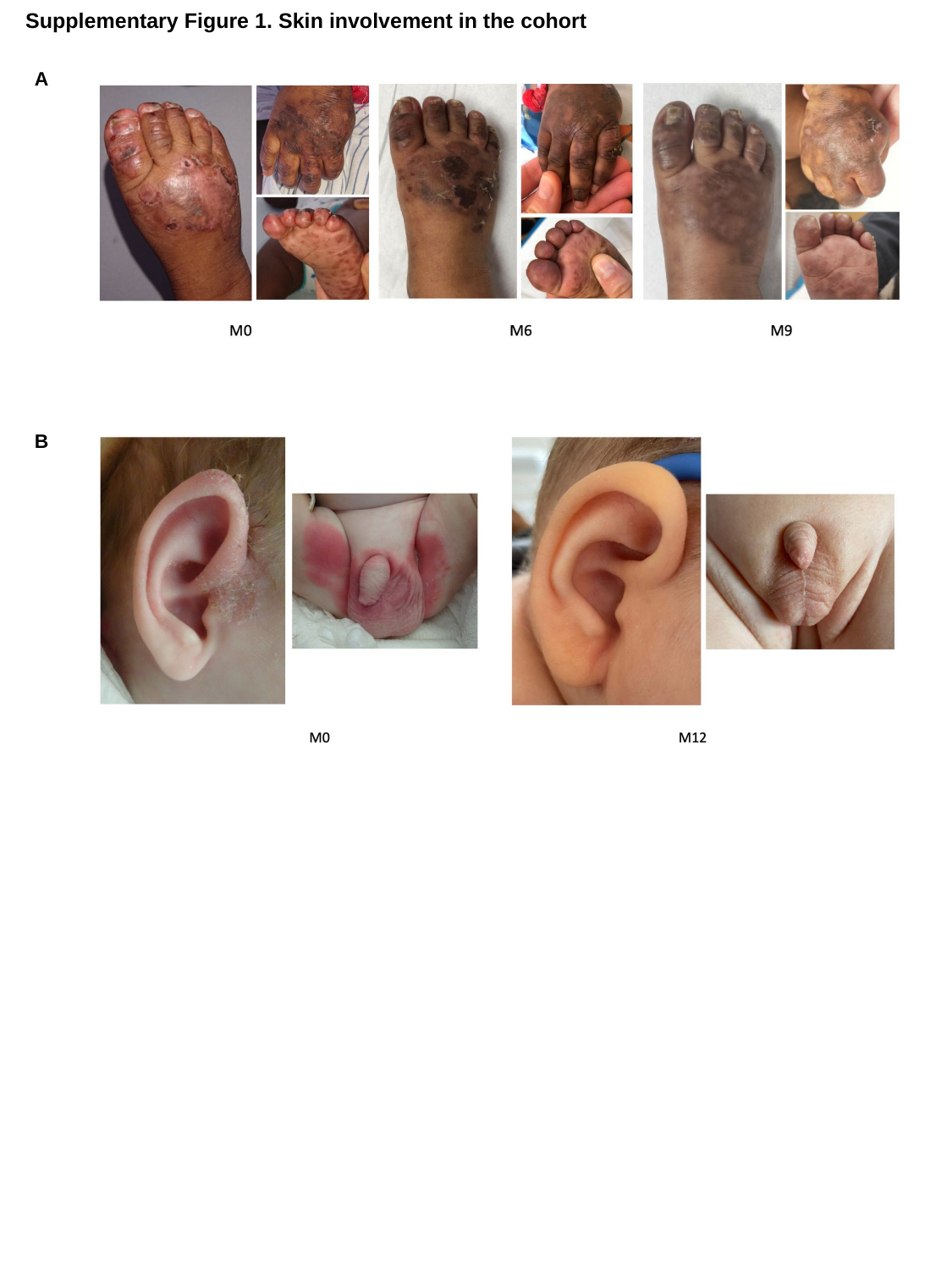

Supplementary Figure 1. Skin involvement in the cohort
A
B

## Slide 2
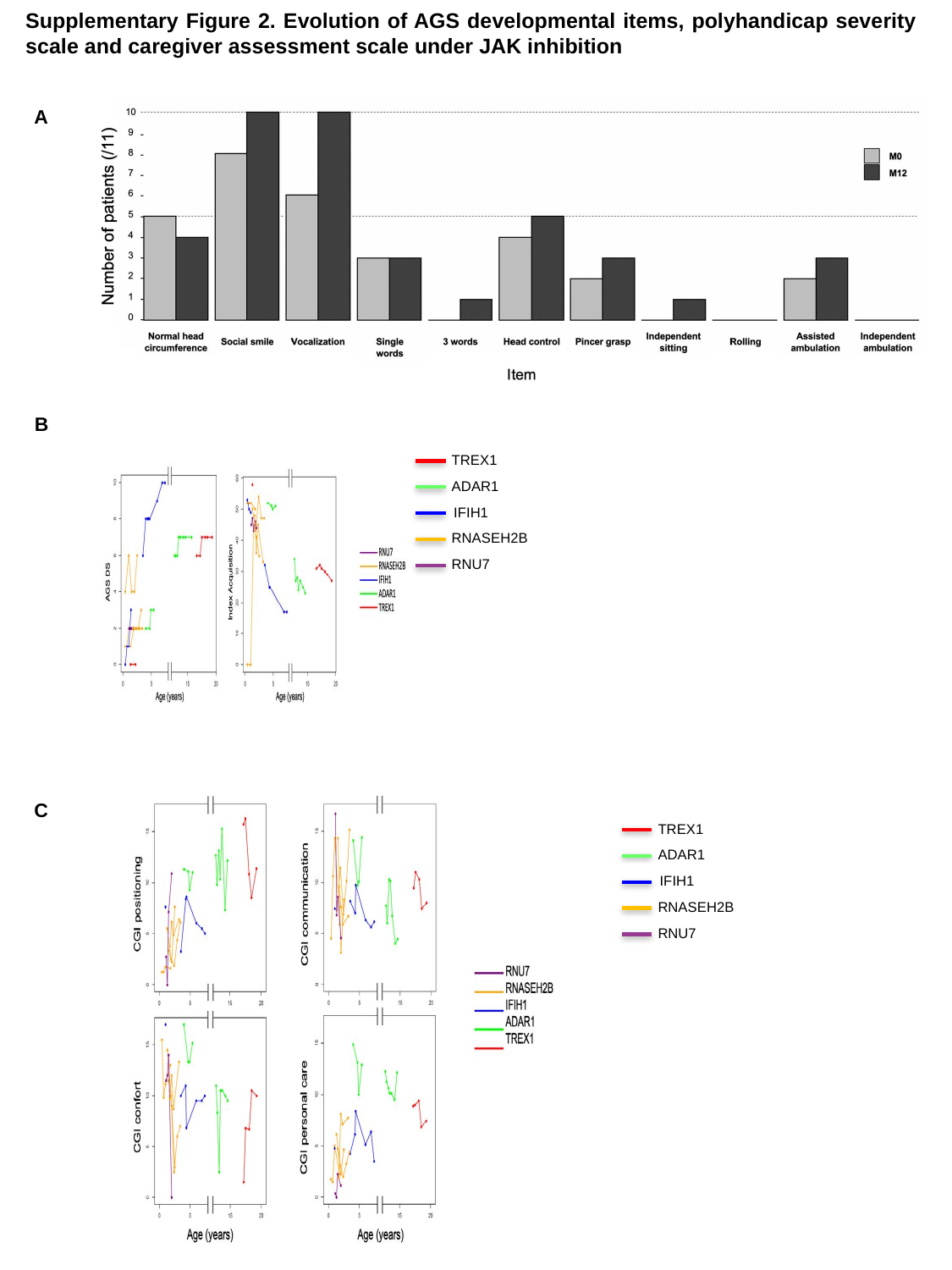

Supplementary Figure 2. Evolution of AGS developmental items, polyhandicap severity scale and caregiver assessment scale under JAK inhibition
A
B
TREX1
ADAR1
IFIH1
RNASEH2B
RNU7
C
TREX1
ADAR1
IFIH1
RNASEH2B
RNU7

## Slide 3
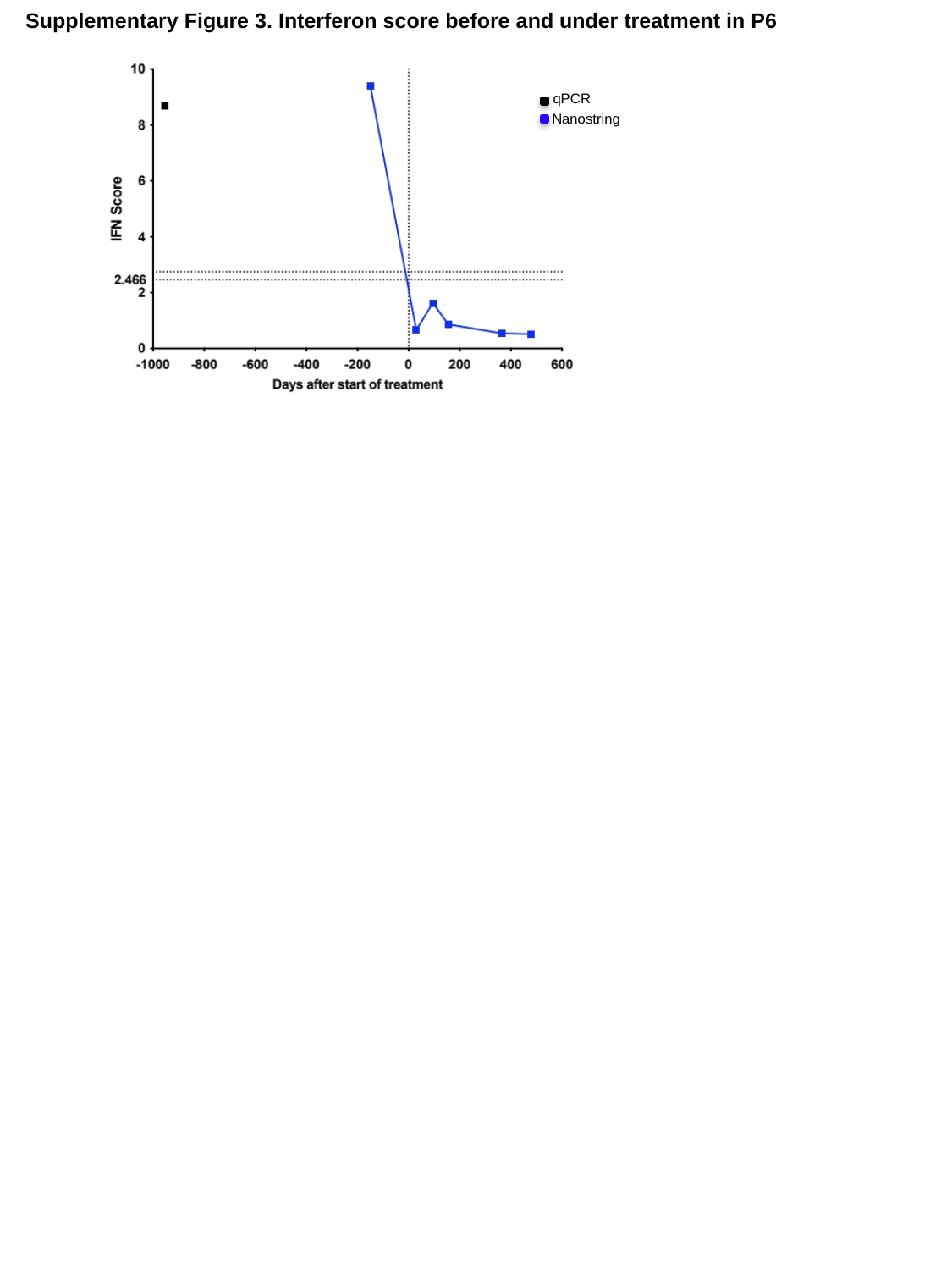

Supplementary Figure 3. Interferon score before and under treatment in P6
qPCR
Nanostring

## Slide 4
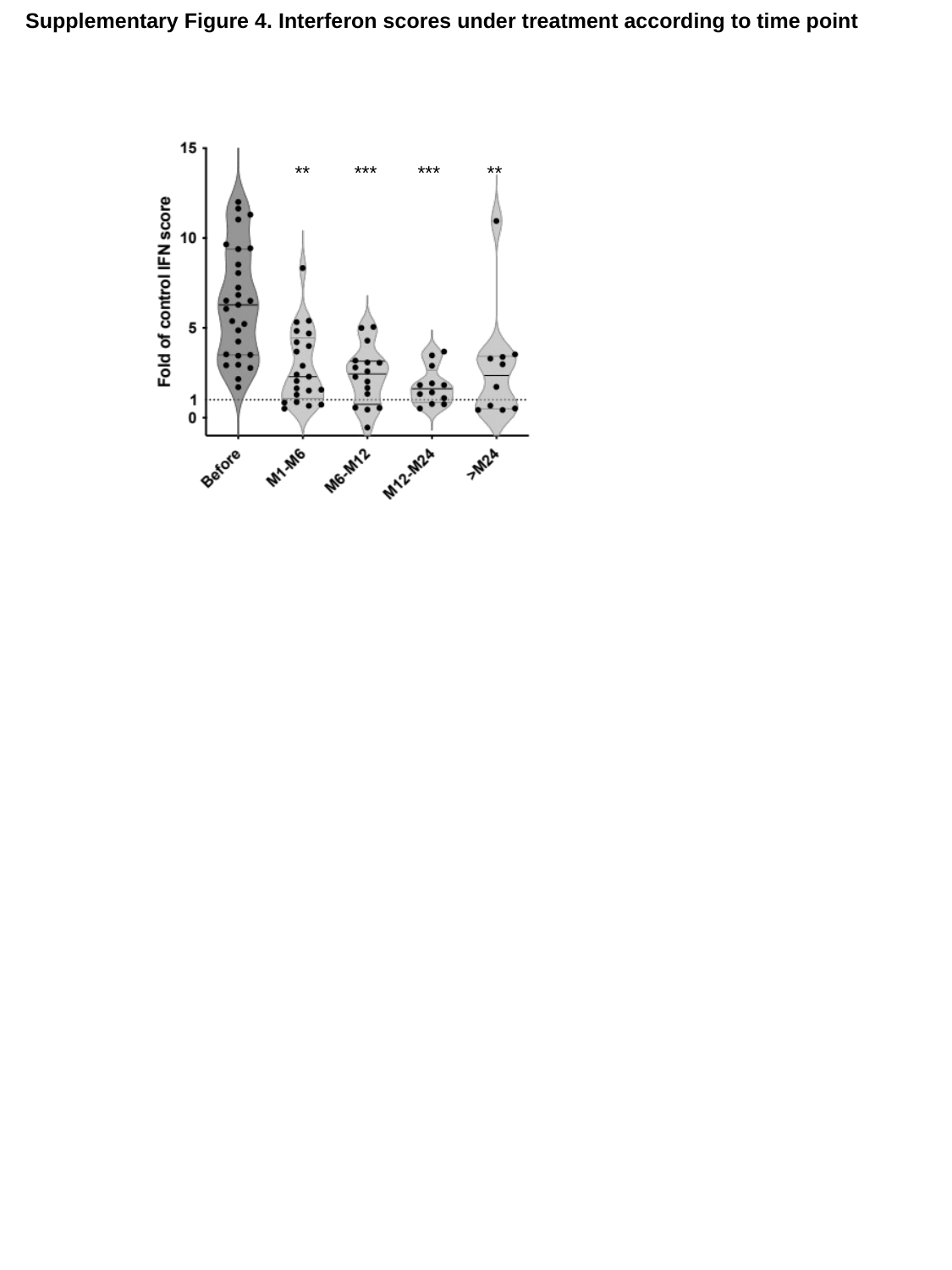

Supplementary Figure 4. Interferon scores under treatment according to time point
**
***
***
**

## Slide 5
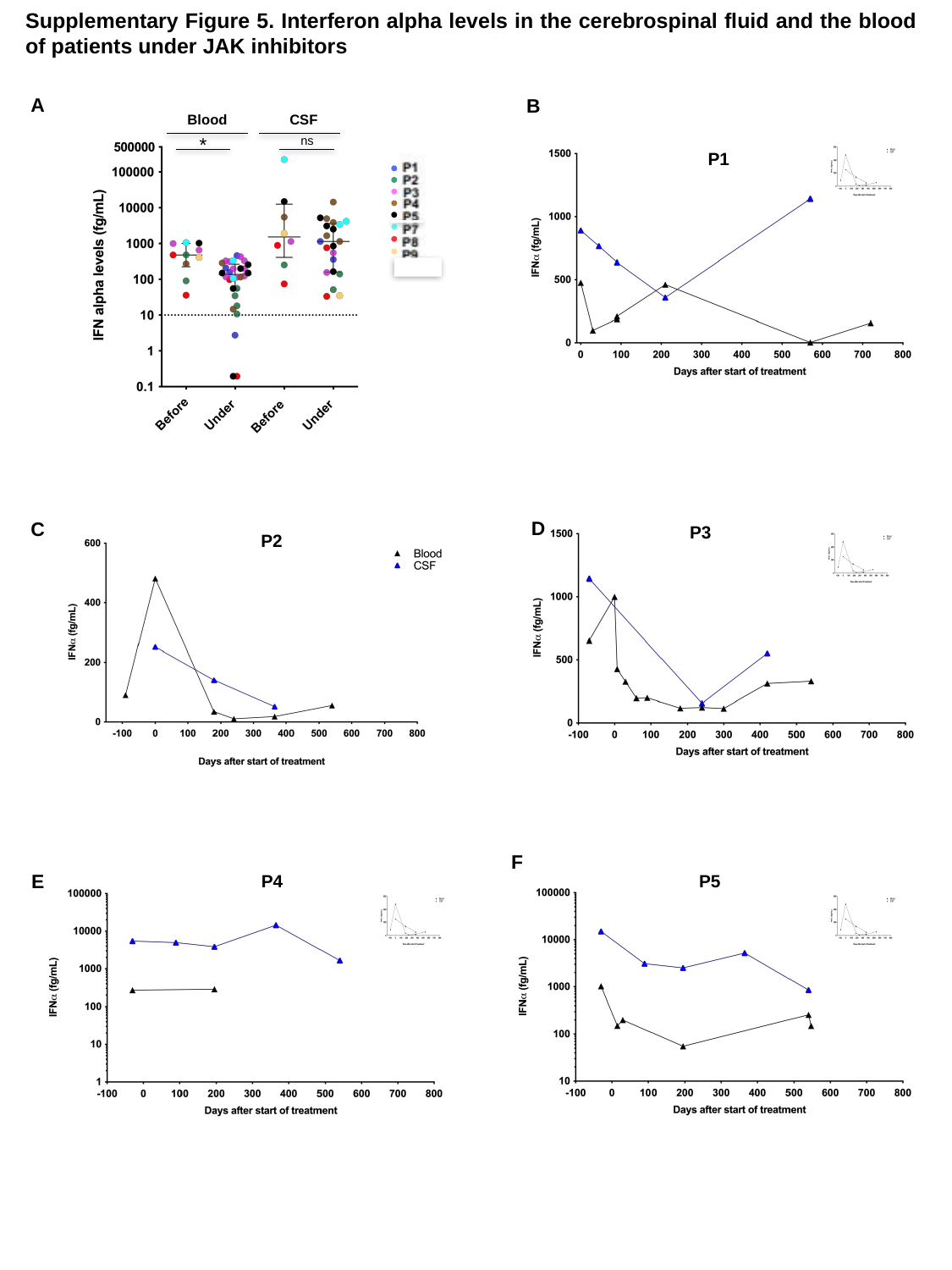

Supplementary Figure 5. Interferon alpha levels in the cerebrospinal fluid and the blood of patients under JAK inhibitors
A
B
Blood
CSF
*
ns
P1
D
C
P3
P2
F
E
P5
P4

## Slide 6
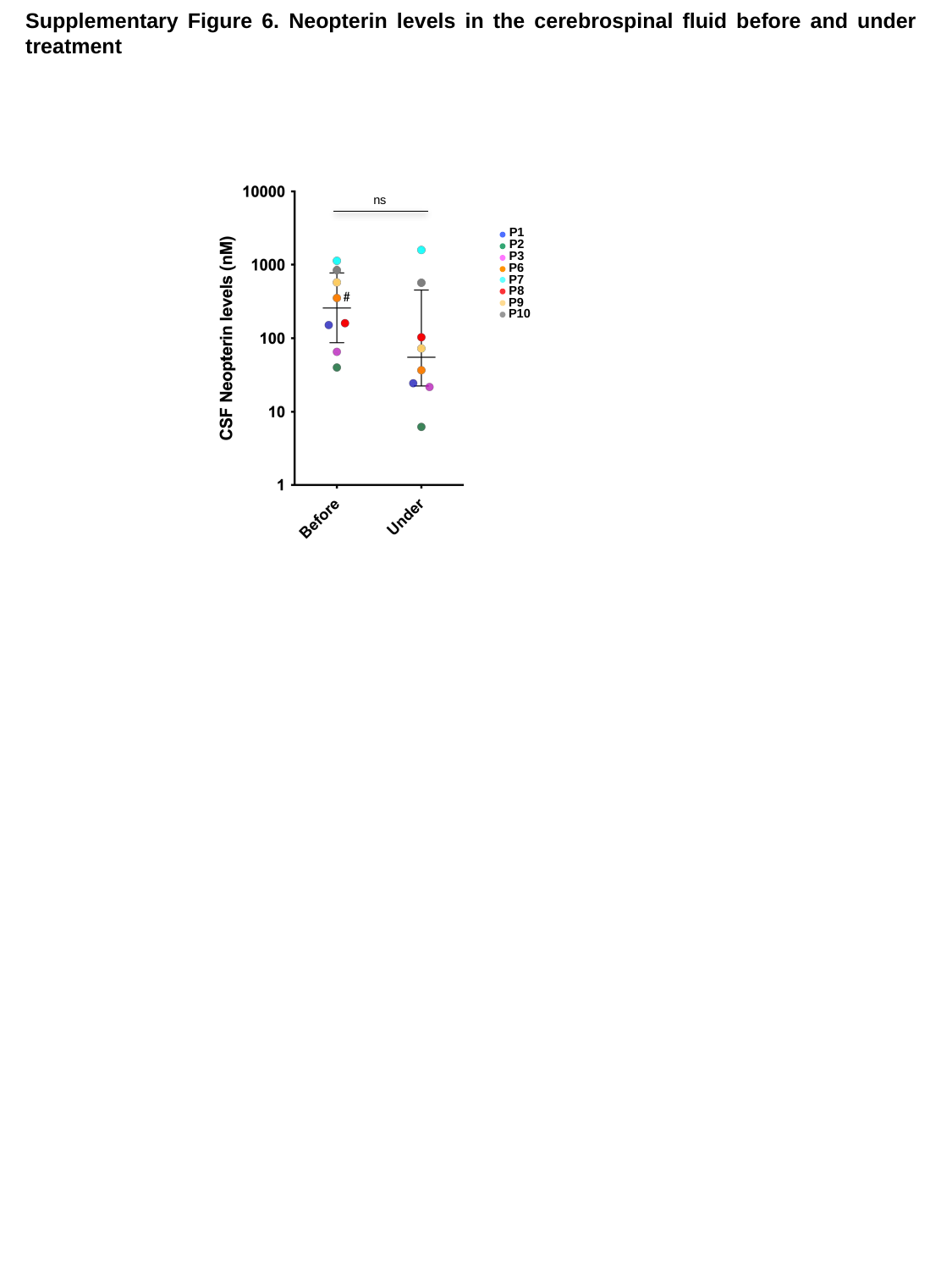

Supplementary Figure 6. Neopterin levels in the cerebrospinal fluid before and under treatment
ns
P1
P2
P3
P6
P7
P8
P9
P10
#

## Slide 7
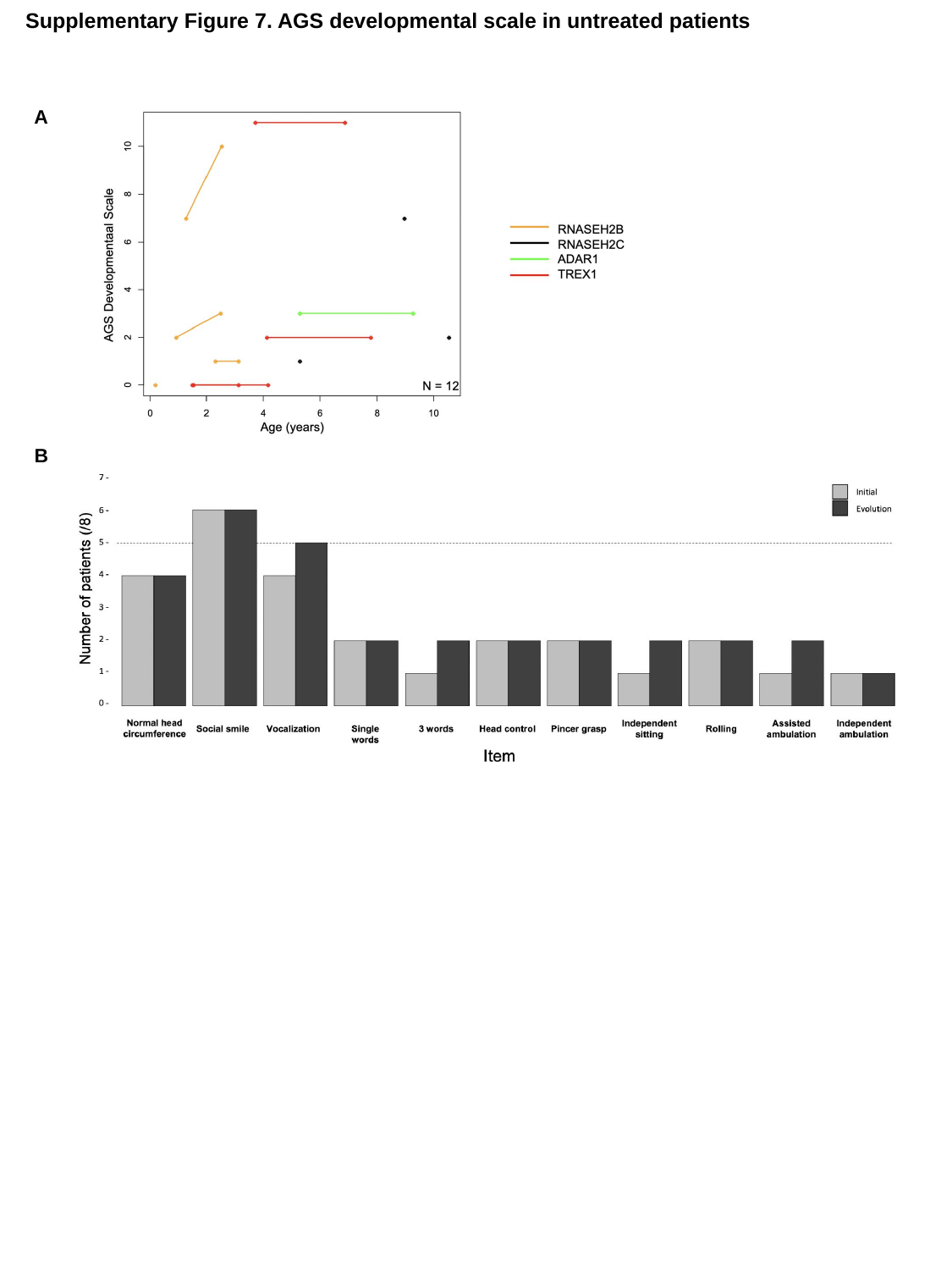

Supplementary Figure 7. AGS developmental scale in untreated patients
A
B
