# Supplementary material for: JAK Inhibition in Aicardi-Goutières Syndrome: a Monocentric Multidisciplinary Real-World Approach Study
Source: J Clin Immunol. 2023 May 12;43(6):1436–47. doi: 10.1007/s10875-023-01500-z (PMC10175907; doi:10.1007/s10875-023-01500-z)
Supplement: Supplementary file 2 — (DOCX 68.7 kb) [file 10875_2023_1500_MOESM2_ESM.docx]

**Supplemental material**

**Supplementary Figures Legends**

**Figure S1. Skin involvement in the cohort**

(A) Chilblain-like ulcerations of the feet and hand of P11 observed before initiation of baricitinib (M0, spring period, left panel), with a partial response after 6 months of treatment (M6, autumn period, middle panel) that had further improved three months later (M9, winter period, right panel).

(B) Psoriatic lesions of the helix, the tragus, the thighs and the external genitalia observed in P10 before initiation of baricitinib (M0, winter period, left panel), with complete remission after 16 months of treatment (M16, spring period, right panel).

**Figure S2. Evolution of AGS developmental items, polyhandicap severity scale and caregiver assessment scale under JAK inhibition**

(A) Evolution of the different items of the AGS neurologic severity scale^14^ (DS) in patients under JAK inhibition between M0 (before treatment) and at M12 (after one year of treatment): the item ‘vocalization’ is the only one demonstrating a statistically significant improvement (p=0.038, paired t-test).

(B) Evolution of the polyhandicap severity scale (index of acquisitions) in patients under JAK inhibition according to age and mutant genotype.

(C) Evolution of the different sub-scales of the Caregiver questionnaire in patients under JAK inhibition according to age and mutant phenotype.

**Figure S3. Interferon score before and under treatment in P6**

Interferon (IFN) score assessed by RT-qPCR^20^ or Nanostring^21^ in the whole blood of P6 before and under treatment with baricitinib.

**Figure S4. Interferon scores under treatment according to time point**

Interferon (IFN) scores assessed by qRT-PCR^20^ or Nanostring^21^ in the whole blood of patients (P1 - P11) before and under treatment with a JAK inhibitor. Data are expressed as fold values of the normal interferon score (i.e. 2.466 and 2.724 for qRT-PCR and Nanostring technologies respectively). Median and interquartile ranges of interferon scores are represented for each follow-up time point in violin plots. Data were analysed statistically using the Kruskall-Wallis test (***p<0.001, **p<0.01, statistics above violin plots).

**Figure S5. Interferon alpha levels in the cerebrospinal fluid and the blood of patients under JAK inhibitors**

(A) Interferon (IFN) alpha protein concentration assessed by Simoa digital ELISA^22^ in serum / plasma (blood) and cerebrospinal fluid (CSF) of patients (P1-P5 and P7-P9) before and under treatment with JAK inhibitors (ruxolitinib). Data were not available for P6, P10 and P11. Horizontal bars indicate median values and interquartile ranges. Comparison among groups was performed with one-way ANOVA test (Kruskal–Wallis) and Dunn’s multiple comparison testing (ns, non-significant, *p<0.05).

(B to F) Concentration of IFN alpha measured by Simoa^22^ in the CSF and the blood of P1 to P5 before and under treatment with a JAK inhibitor.

**Figure S6. Neopterin levels in the cerebrospinal fluid before and under treatment**

Neopterin levels measured by LC-MSMS in the cerebrospinal fluid (CSF) of patients before (n = 8 samples) and under treatment (n = 8 samples) with baricitinib. Comparison between the two groups was performed using Mann-Whitney test (ns, not significant). # indicates one sample taken in P6 three years before JAK inhibitor initiation.

**Figure S7. AGS developmental scale in untreated patients**

(A) AGS developmental scale^14^ in patients untreated at screening (12 out 12 patients) and during the natural course of their disease (8 out 12 patients).

(B) Different items of the AGS developmental scale^14^ available from untreated patients for which follow-up is available (8 out 12 patients).

**Table S1. Cerebrospinal fluid parameters at screening (clear background) and under treatment (grey background)**

|  | **AGS number** | **Genotype** | **Age at LP (y)** | **Follow-up (m)** | **Number of WBCs** | **Number of RBCs** | **CSF histology** | **CSF protein level (g/L)** | **Blood protein level (g/L)** | **CSF IFN alpha level* (fg/mL)** | **Blood IFN alpha level* (fg/mL)** | **CSF neopterins**^§^ **(nmol/L)** |
| --- | --- | --- | --- | --- | --- | --- | --- | --- | --- | --- | --- | --- |
| **P1** | AGS0769 | TREX1 | 16.56 | Screening | 0 | 163 | Haemorrhagic | 0.21 | 71 | 890.19 | 476.53 | 152.2 |
|  |  |  |  | 19 | 0 | 1 | Acellular | 0.23 | 78 | 1141.49 | **2.76** | **24.4** |
| **P2** | AGS1537 | ADAR1 | 12.70 | Screening | 0 | 1 | Few cells, predominance of mature ly | 0.29 | 77 | 251.66 | 481.34 | 40.2 |
|  |  |  | 13.73 | 12 | 2 | 16 | Few cells, predominance of mature ly | 0.24 | 76 | **51.60** | **18.19** | **6.2** |
| **P3** | AGS2180 | IFIH1 | 3.50 | Screening | 0 | 0 | Acellular | 0.16 | 83 | 1144.00 | 652.73 | 65.2 |
|  |  |  | 4.81 | 14 | 1 | 12 | Few cells, predominance of mature ly | 0.15 | 77 | **551.81** | **311.76** | **21.7** |
| **P4** | AGS2437.1 | RNASEH2B | 1.93 | Screening | 7 | +++ | Haemorrhagic | 0.86 | 69 | 5446.93 | 270.28 | NA |
|  |  |  | 4.07 | 24 | 5 | 0 | Very few cells, with scarce ly | 0.19 | 72 | **1141.93** | **14.59** | 40.5 |
| **P5** | AGS2437.2 | RNASEH2B | 0.36 | Screening | 7 | 0 | High cellularity with predominance of mature ly | 0.36 | 68 | 3091.32 | 1025.1 | NA |
|  |  |  | 2.5 | 24 | 2 | 0 | No cell | 0.18 | NA | **164.16** | **0.19** | 71.2 |
| **P6** | AGS2350 | ADAR1 | 1.2 | 3y before screening | NA | NA | NA | NA | NA | NA | NA | 353** |
|  |  |  |  | NA | / | / | / | / | / | / | / | / |
| **P7** | AGS2931 | RNASEH2B | 0.36 | Screening | 50 (100% ly) | 4 | Macrophages, mature ly | 1.78 | 63 | 221614.57 | 1063.92 | 1130.4 |
|  |  |  | 1.75 | 16 | 0 | 0 | NA | 0.18 | 78 | **4162.34** | **333.62** | 1196 |
| **P8** | AGS2895 | RNU7 | 1.07 | Screening | 2 | 0 | Few cells, predominance of mature ly | 0.46 | 69 | 74.51 | 35.75 | 161 |
|  |  |  | 2.12 | 12 | 1 | 0 | Very few cells, with scarce ly | 0.25 | 78 | **33.42** | **0.19** | **103.5** |
| **P9** | AGS3031 | RNASEH2B | 1.69 | Screening | 1 | 0 | Mild inflammation, predominance of mature ly | 0.22 | 74 | 1907 | 409.745 | 579 |
|  |  |  |  | NA | / | / | / | / | / | / | / | / |
| **P10** | AGS3065 | IFIH1 | 0.39 | Screening | 14 (84% ly) | 110 | NA | 1.10 | 73 | NA | NA | 850 |
|  |  |  |  | NA | / | / | / | / | / | / | / | / |
| **P11** | AGS2831 | TREX1 | 0.02 | Screening | 64 | 0 | Inflammation, predominance of mature ly | 0.95 | NA | NA | NA | NA |
|  |  |  |  | NA | / | / | / | / | / | / | / | / |

**Abbreviations**: CSF: cerebrospinal fluid; IFN: interferon; LP: lumbar puncture; ly: lymphocytes; NA: not assessed; RBCs: red blood cells; y: years; WBCs; white blood cells.

*Using anti-IFN pan-alpha antibody as reported in^22^. Normal values below 10 fg/mL. Decreased values under treatment are indicated in bold.

^§^Normal values between 9 and 34 nmol/L. Decreased values under treatment are indicated in bold.

**Table S2. AGS patients evaluated but not treated with JAK inhibitors**

|  | **AGS number** | **Genotype** | **Mutation** | **Sex** | **Age at onset (m)** | **Age at diagnosis (m)** | **Age at evaluation (m)** | **CNS features** | **Skin features** | **Other** | **Reason for not treating** | **Age at last status (m)** | **Last status** |
| --- | --- | --- | --- | --- | --- | --- | --- | --- | --- | --- | --- | --- | --- |
| **P12** | AGS2545 | TREX1 | p.Arg114His, p.Ser82Leufs*9 | F | antenatal | 3 | 11 | Dystonic-spastic tetraparesis, axial hypotonia | Very mild chilblain | Cardiomyopathy, transient thrombocytopenia and cholestasis | Cardiomyopathy and severe brain damage | 50 | Alive |
| **P13** | AGS2397 | TREX1 | p.Arg114His Ho | M | antenatal | 7 | 19 | Epilepsy, dystonic tetraparesis, axial hypotonia, discomfort | Mild urticaria at 6m | Transient thrombocytopenia and transaminitis | Severe brain damage | 39 | Deceased |
| **P14** | AGS2938 | RNASEH2B | p.Ala177Thr, p.I220N | F | antenatal | 1.5 | 2.5 | Spastic-dystonic tetraparesis, axial hypotonia, discomfort | None | None | Severe brain damage | 2* | Alive |
| **P15** | AGS0788 | ADAR1 | p.Pro193Ala, p.Asp462Glnfs*2 | F | 15 | 27 | 67 | Spastic-dystonic tetraparesis | None | None | Parental decision | 113 | Alive |
| **P16** | AGS2499 | TREX1 | p.Arg62His, p.Cys99Metfs*3 | M | 12 | 36 | 40 | Very mild pyramidal syndrome, cerebral MRI and CT Scan normal | Mild chilblains in winter | none | Minimal skin and neurological involvement  Parental decision | 84 | Alive |
| **P17** | AGS3078 | RNASEH2B | p.Ala177Thr, p.Lys162Thr | F | 1 to 6 | 8 | 12 | Delay of Myelinisation, intracranial calcification.  Spastic-dystonic tetraparesis, axial hypotonia, epileptic spasms | None | None | Parental decision | 30 | Alive |
| **P18** | AGS2916 | RNASEH2B | p.Ala177Thr Ho | F | 4 | 24 | 25 | Spastic-dystonic tetraparesis  Good interaction | None | None | Normal CSF, myelinisation in progression, no discomfort | 31 | Alive |
| **P19** | AGS0958 | TREX1 | p.Arg114His, p.Thr303Pro | F | 2 | 8 | NR | Spastic-dystonic tetraparesis, intracranial calcification | None | None | Fixed lesions | 95 | Alive |
| **P20** | AGS2892 | RNASEH2B | p.Ala177Thr Ho | M | 6 | 8 | 26 | Spastic-dystonic tetraparesis | None | None | Parental decision | 38 | Alive |
| **P21** | AGS3058.1 | RNASEH2C | p.Arg145Cys Ho | M | 8 | 107 | 108 | Spastic-dystonic tetraparesis, intracranial calcification | None | Possible deforming arthropathy | Severe brain damage, no discomfort | 109* | Alive |
| **P22** | AGS3058.2 | RNASEH2C | p.Arg145Cys Ho | M | 7 | 125 | 126 | Spastic-dystonic tetraparesis, intracranial calcification | None | None | Severe brain damage, no discomfort | 128* | Alive |
| **P23** | AGS3058.3 | RNASEH2C | p.Arg145Cys Ho | M | 6-8 | 62 | 63 | Spastic-dystonic tetraparesis, intracranial calcification | None | None | Severe brain damage, no discomfort | 64* | Alive |

**Abbreviations**: CT: computed tomography; F: female; M: male; m: months; MRI: magnetic resonance imaging; NR: not recorded; P: patient.

*For these patients, further follow-up after the initial clinical extended evaluation was not available.

**Table S3. Cerebrospinal fluid parameters at evaluation in untreated patients**

|  | **AGS number** | **Genotype** | **Age at LP in y (during screening)** | **Number of WBCs** | **Number of RBCs** | **CSF histology** | **CSF protein level (g/L)** | **Blood protein level (g/L)** | **CSF IFN alpha level*** **(fg/mL)** | **Blood IFN alpha level*** **(fg/mL)** | **CSF neopterins**^§^ **(nmol/L)** |
| --- | --- | --- | --- | --- | --- | --- | --- | --- | --- | --- | --- |
| **P12** | AGS2545 | TREX1 | 0.41 | NA | NA | NA | 1.11 | 69 | NA | NA | NA |
| **P13** | AGS2397 | TREX1 | NA |  |  |  |  |  |  |  |  |
| **P14** | AGS2938 | RNASEH2B | 0.20 | 27 | 0 | Hypercellularity, predominance of mature ly, some activated cells | 0.51 | 61 | NA | NA | 1055 |
| **P15** | AGS0788 | ADAR1 | 1.30 | 2 | 0 | NA | 0.19 | 61 | NA | NA | NA |
| **P16** | AGS2499 | TREX1 | NA |  |  |  |  |  |  |  |  |
| **P17** | AGS3078 | RNASEH2B | 0.92 | 12 | 2 | Mild cellularity, predominance of activated ly | 0.34 | 66 | ongoing | Ongoing | NA |
| **P18** | AGS2916 | RNASEH2B | 2.22 | 0 | 0 | No cellularity | 0.11 | 72 | ongoing | Ongoing | NA |
| **P19** | AGS0958 | TREX1 | NA |  |  |  |  |  |  |  |  |
| **P20** | AGS2892 | RNASEH2B | 0.67 | 21 | 4 | Hypercellularity, predominance of ly | 0.42 | 63 | NA | NA | NA |
| **P21** | AGS3058.1 | RNASEH2C | NA |  |  |  |  |  |  |  |  |
| **P22** | AGS3058.2 | RNASEH2C | NA |  |  |  |  |  |  |  |  |
| **P23** | AGS3058.3 | RNASEH2C | NA |  |  |  |  |  |  |  |  |

**Abbreviations**: CSF: cerebrospinal fluid; IFN: interferon; LP: lumbar puncture; ly: lymphocytes; NA: not assessed; P: patient; RBCs: red blood cells; y: years; WBCs; white blood cells.

*Using anti-interferon pan-alpha antibody as reported in^22^. Normal values below 10 fg/m.

^§^Normal values between 9 and 34 nmol/L.

**Table S4. Baricitinib concentration in serum**

| **Patient** | **AGS number** | **Follow-up (m)** | **Baricitinib AUC0-24h (ng*h/mL)** |
| --- | --- | --- | --- |
| **P6** | AGS2350 | 6 | 1172 |
|  |  | 11 | 1181 |
| **P7** | AGS2931 | 3 | 1128 |
| **P8** | AGS2895 | 11 | 1555 |
| **P9** | AGS3031 | 3 | 1290 |
| **P10** | AGS3065 | 1 | 1446 |
|  |  | 3 | 1335 |
|  |  | 6 | 1443 |
|  |  | 15 | 1332 |
| **Median** | / | **6** | **1332** |

**Abbreviations**: m: month; P: patient.

**Table S5. Baricitinib concentration in cerebrospinal fluid**

| **Patient** | **AGS number** | **Follow-up (m)** | **Baricitinib CSF concentration (ng/mL)** |
| --- | --- | --- | --- |
| **P6** | AGS2350 | 15 | 10.6 |
| **P7** | AGS2931 | 3 | 6.5 |
|  |  | 8 | 13.6 |
|  |  | 16 | 11.1 |
| **P8** | AGS2895 | 1 | 10.2 |
|  |  | 7.26 | 7.3 |
| **P9** | AGS3031 | 3 | 6.3 |
|  |  | 6 | 6.4 |
|  |  | 7 | 1.2 |
|  |  | 12 | 1.7 |
| **P10** | AGS3065 | 6 | 15.3 |
| **Median** | / | **6** | **7.3** |

**Abbreviations**: CSF: cerebrospinal fluid; m: month; P: patient.
